# Effective Treatment of Bovine Mastitis with Intramammary Infusion of* Angelica dahurica* and* Rheum officinale* Extracts

**DOI:** 10.1155/2019/7242705

**Published:** 2019-03-03

**Authors:** Wan-Ting Yang, Chun-Yen Ke, Wen-Tien Wu, Ru-Ping Lee, Yi-Hsiung Tseng

**Affiliations:** ^1^Institute of Medical Sciences, Tzu Chi University, Hualien 97004, Taiwan; ^2^Department of Nursing, St. Mary's Medicine, Nursing and Management College, Yi-Lan 26644, Taiwan; ^3^Department of Nursing, Tzu Chi University of Science and Technology, Hualien 970, Taiwan; ^4^Department of Orthopedics, Hualien Tzu Chi Hospital, Buddhist Tzu Chi Medical Foundation, Hualien 97002, Taiwan; ^5^School of Medicine, Tzu Chi University, Hualien 97004, Taiwan; ^6^Institute of Molecular Biology, National Chung Hsing University, Taichung 402, Taiwan

## Abstract

Mastitis in dairy cattle is a highly prevalent infectious disease, causing considerable economic loss worldwide. In this study, we used* Angelica dahurica* and* Rheum officinale* extracts (designated as Yi-Xiong-Tang, YXT) for mastitis treatment. California mastitis test (CMT) was performed and 67 mastitis udder quarters were identified among 179 lactating dairy cows. These 67 mastitis udder quarters were subjected to treatments by intramammary infusion of YXT twice a day for three consecutive days. The mastitis indicators including clots, lactate dehydrogenase (LDH), TNF-*α*, IL-6, IL-8, and total viable count of bacteria (TVC) in milk were examined before and after the YXT treatment to evaluate its effectiveness. Levels of mastitis indicators from mastitis udder quarters were elevated. After YXT treatment, normal levels of these indicators were restored: TVC, 2.10 × 10^4^ – 9.20 × 10^6^ CFU/mL; clots, 6.56 ± 0.43 mg/mL; LDH, 181.0 ± 18.55 U/L; TNF-*α*, 0.02 ± 0.02 ng/mL; IL-6, 41.4 ± 11.46 pg/mL; and IL-8, 1.85 ± 0.60 pg/mL. Compared with the antibiotic therapy, YXT treatment has a shorter treatment course and might have lower probability for the causative agents to develop drug resistance because YXT is in fact a cocktail containing multiple active ingredients.

## 1. Introduction

Bovine mastitis is an infectious disease of the mammary glands of dairy cattle, with high worldwide prevalence at both the cow and udder quarter levels [1–3]. Cows with mastitis cannot be milked regularly after the mammary gland cells are damaged. In addition, clot formation is common in mastitis milk; this is caused by enzymes produced by the pathogenic bacteria, such as coagulase, which enable the conversion of fibrinogen to fibrin [4, 5]. Collectively, these factors alter milk quality, reduce milk yield, and increase treatment and care costs, resulting in great economic losses to the dairy industry [6, 7].

The primary mastitis-causing pathogens include contagious microorganisms that survive and proliferate on the skin and teat wounds, e.g.,* Streptococcus agalactiae*,* Staphylococcus aureus*, and* Strep. dysgalactiae*, as well as environmental microorganisms that are not retained on the teat, e.g.,* Strep. uberis*,* Escherichia coli*, and other coliforms [8–10]. These bacteria enter mammary glands of cows through their teat canal, where they colonize, proliferate, and release toxins, damaging the mammary gland cells [11, 12]. An increase in lactic dehydrogenase (LDH) activity in milk is an indication of the infection [13, 14]. Immediately after infection, inflammatory responses are elicited; for instance, the number of somatic cells and levels of inflammatory cytokines, such as tumor necrosis factor (TNF)-*α*, interleukin (IL)-6, and IL-8, increase in milk [15–18].

Detection of mastitis is generally based on indicators of the inflammation, such as somatic cell count (SCC), inflammatory cytokine, enzyme activity (e.g., LDH or NAGase), and the electrical conductivity [19]. However, California Mastitis Test (CMT) is widely used for preliminary detection of bovine mastitis, which is a cost effective, rapid, user friendly method used to measure the SCC in milk samples [19].

The main treatment of mastitis is commonly administered by intramammary infusion of an ointment or intramuscular or intravenous injection of antibiotics, such as streptomycin, ampicillin, cloxacillin, penicillin, and tetracycline [8]. However, the treatment is anticipated to become problematic in the near future owing to the rapid increase in antibiotic-resistant pathogens [20, 21]. Therefore, seeking for treatments alternative to antibiotic therapy is required. Traditional Chinese medicine (TCM) for bovine mastitis treatment is commonly prepared from* Taraxacum mongolicum* (Pu Gong Ying),* Lonicera japonica* Thunb. (Jin Yin Hua),* Viola patrinu* (Zi Hua Di Ding), and* Folium isatidis* (Da Qing Ye), based on their heat-clearing, detoxifying, anti-inflammatory, and antibacterial actions, which are orally administered [22]. Many other TCM herbs possess similar pharmacological effects, such as* Angelica dahurica *(Bai Zhi),* Coptis chinensis *(Huang Lian),* Phellodendron amurense* (Huang Bo),* Rheum officinale* (Da Huang), and* Scutellaria baicalensis* (Huang Qin) [23, 24]. These herbs, being “cold (寒, hán)” according to the TCM concept, which can clear away internal heat and are generally used as antibiotic and antipyretic agents, are thus considered to have anti-inflammatory and antimicrobial effects and be effective in treating inflammatory diseases and microbial infections [24].

In our previous study,* A. dahurica* and* R. officinale *extracts, designated as Yi-Xiong-Tang (YXT), were found to have antimicrobial, anti-inflammatory, and wound healing effects [25]. However, its application in bovine mastitis treatment has not been studied. In this study, we investigated the therapeutic effectiveness of YXT in cows with mastitis in a dairy farm in eastern Taiwan and the results are described in this report.

## 2. Materials and Methods

### 2.1. YXT Preparation

The* A. dahurica *and* R. officinale *extracts, previously called ARE, were prepared as described previously, with the detailed description of the preparation and content of the extracts being given by Yang et al., 2017 [25]. Briefly, 100 g of each powder of* A. dahurica* and* R. officinale* (Hou-Chuia Biopharm Co., Ltd., Tainan City, Taiwan) was individually added to 800 mL of 70% ethanol, and the mixture was heated at 70°C for 24 hour. The extracts were centrifuged at 10,000 × g for 15 minutes, and the supernatants were concentrated under reduced pressure to remove the whole ethanol. This extract was designated as YXT here.

### 2.2. Quantification of the Active Constituents in YXT

Quantification of the five possible active constituents in YXT, emodin, rhein, furanocoumarin, imperatorin, and polysaccharides, was provided by Herbiotek Co., Ltd., New Taipei City, Taiwan. Twenty milliliter of YXT was filtered through 0.22-*μ*m membranes before analyzed. The Waters HPLC system (Milford, Massachusetts, USA) comprised Waters 600 pump system, Waters 2996 Photodiode array detector, Waters 717 plus Autosampler, and Sugai U-620 Column oven (Wakayama City, Japan). Cosmosil 5C18-MS-II reversed phase column (5 *μ*m, 4.6 mm × 250 mm, Nacalai Tesque, Japan) equipped with LiChrospher RP-18 end-capped guard column (5 *μ*m, 4.0 mm × 10 mm, Merck, Germany) was used as the stationary phase. The gradient elution was composed of eluents A, B, and C (A: H_2_O/KH_2_PO_4_/10% H_3_PO_4_ = 1000 mL/2.72 g/1 mL; B: acetonitrile; C: H_2_O) with the following profile: 0-20 min, 70%-40% A and 30%-60% B; 20-30 min, 40%-0% A, 60%-80% B, and 0%-20% C; 30-35 min, 80%-30% B and 20%-70% C; 35-40 min, 0%-70% A, 30% B, and 70%-0% C. The flow rate was 1 mL/min, and the column temperature was maintained at 35°C. UV 270 nm was used for detection of rhein and emodin, with a retention time of 17.1 and 27.7 min, respectively. UV 300 nm was used for detection of imperatorin and isoimperatorin (derivatives of furanocoumarin), with a retention time of 26.7 and 29.8 min, respectively. Polysaccharides in YXT were determined according to the Pharmacopoeia of the People's Republic of China 2015 (pp. 188-189), absorbance was read at 625 nm in a PRO-7990 UV-Vis spectrophotometer (PREMA Products, Inc., GA, USA).

### 2.3. Animals and Treatment

The lactating dairy cows (Holstein cows) in the ranch (Ji-Zheng ranch, Hualien, Taiwan) were the test subjects in this study. A total of 716 udder quarters (from 179 lactating dairy cows) were examined using the California Mastitis Test (CMT) to detect mastitis and identify the affected udder quarters. The mastitis udder quarters with CMT scored 2 and 3 were administered YXT (10 mL) through intramammary infusion into the teat canals twice a day, immediately after routine milking, for 3 consecutive days. All experiments were performed under institutionally approved protocols provided by the Animal Care and Use Committee of Tzu Chi University approved the experimental protocol (No: 103078).

### 2.4. Milk Sample Collection and Analysis

Milk samples were collected before and after the 3-day YXT treatment. The udders of the subjects were washed with abundant clean water and wiped with paper towels, and the teats were surface-disinfected with swabs containing 75% ethanol. After discarding the first stream, approximately 40 mL of quarter milk was collected into 50-mL sterilized tubes and stored on ice for immediate examination on delivery to our laboratory. All milk samples were divided into aliquots for the following tests:

#### 2.4.1. CMT

CMT was performed by using an ImmuCell California Mastitis Test Kit (Portland, ME, USA). For each test, 5 mL of milk was added into a testing paddle and mixed with 5 mL CMT solution, followed by gentle rotation of the mixture in the horizontal position. The reaction was visualized within 30 s for determining the somatic cell counts (SCC), according to manufacturer instructions. The reactions were scored as N, T, 1, 2, and 3, representing negative (less than 200,000 cells/mL), trace (200,000–500,000 cells/mL), weakly positive (500,000–1,500,000 cells/mL), distinctly positive (1,500,000–5,000,000 cells/mL), and strongly positive (more than 5,000,000 cells/mL) results for mastitis, respectively.

#### 2.4.2. Microbiological Analysis of Milk

Milk samples were stored at -80°C until used. Bacterial DNA from milk samples was isolated using the EasyPrep Stool Genomic DNA Kit (Biotools Inc., Taipei, Taiwan) according to the manufacturer's instructions. The DNA was used as the templates for PCR amplification of the variable region 4 (V4) of 16S ribosomal RNA gene, using the specific primer set 515F-806R (GTGCCAGCMGCCGCGGTAA and GGACTACHVGGGTWTCTAAT) [26].

The PCR products were subjected to gel electrophoresis (2% agarose) for quality check and then purified with Qiagen Gel Extraction Kit (Qiagen, Germany). Sequencing libraries were generated using TruSeq® DNA PCR-Free Sample Preparation Kit (Illumina, USA) following manufacturer's recommendations, and index codes were added. The library quality was assessed on the Qubit@ 2.0 Fluorometer (Thermo Scientific) and Agilent Bioanalyzer 2100 system. The library was then sequenced on an Illumina HiSeq 2500 platform and 250 bp paired-end reads were generated.

#### 2.4.3. Total Viable Count in Milk

Milk samples were serially diluted and spread onto tryptic soy agar (TSA) plates. After 24–48 h of incubation at 37°C, total colonies on the agar plates were counted. The numbers of colonies were multiplied by dilution factor, converted to the colony forming unit (CFU), and referred to as the total viable count (TVC).

#### 2.4.4. Amount of Clot Formation in Milk

To detect clots in milk, 10 mL of milk was filtered through a 6-*μ*m pore size filter paper (Advantec Grade No. 1 Qualitative Filter Paper). After filtration, the clots retained on filter papers were air-dried and weighed.

#### 2.4.5. LDH and Cytokine Levels in Whey

To measure LDH, TNF-*α*, IL-6, and IL-8 levels, each milk sample was centrifuged at 13,000 ×* g* for 25 min at 4°C to obtain the whey. LDH activity was measured in a Roche Cobas Integra 800 biochemistry analyzer (F. Hoffmann-La Roche Ltd., Basel, Switzerland). TNF-*α*, IL-6, and IL-8 levels were measured using enzyme-linked immunosorbent assay kits from RayBiotech, Inc. (Norcross, GA, USA) and Cloud-Clone Corp. (Houston, TX, USA).

### 2.5. Statistical Analysis

Data were expressed as means ± standard errors. These data were analyzed using the* t* test with SPSS (IBM, NJ, USA), and significant differences were set at* P *< 0.05 (*∗*).

## 3. Results

### 3.1. Emodin, Rhein, and Polysaccharides Were Detected in YXT

HPLC analysis showed that the YXT used in this study contained 30 *μ*g/mL and 80 *μ*g/mL of emodin and rhein, respectively, while furanocoumarin and imperatorin were not detected. Results also showed that the concentration of polysaccharides was 1.08 mg/mL in the YXT used. These compounds have been shown to exhibit antibacterial activities [27, 28].

### 3.2. Mastitis is Highly Prevalent in Dairy Cows

In this study, of the 716 studied udder quarters (from 179 lactating dairy cows), 23 (3.21%) were blind (nonfunctional); the remaining 693 (96.79%) functional quarters were examined using CMT. The results showed that milk from 505 udder quarters (72.87%) was negative for mastitis (non-mastitis); furthermore, 18 udder quarters (2.6%) had possible inflammation (trace in mastitis), 103 (14.86%) had weak mastitis (score 1), 39 (5.63%) had distinct mastitis (score 2), and 28 (4.04%) had strong mastitis (score 3). Thus, the prevalence of mastitis was 54.19% (97/179) at the cow level and 24.53% (170/693) at the udder quarter level. The 16S ribosomal DNA sequencing results showed that the mastitis milk indeed contained the pathogenic bacteria found in mastitis milk [8–10], which included* Streptococcus* spp.,* Streptococcus dysgalactiae*,* Escherichia *spp., and* Staphylococcus *spp. Notably, the amount of* S. dysgalactiae* detected in mastitis milk was 14 times higher than that in non-mastitis milk, suggesting that this species was likely the major pathogen in our mastitis cases.

For subsequent experiments, the udder quarters with distinct or strong mastitis (n = 67) were subjected to YXT treatment, as described in Materials and Methods.

### 3.3. TVC Was Reduced to Normal Levels after YXT Treatment

**Figure 1:**
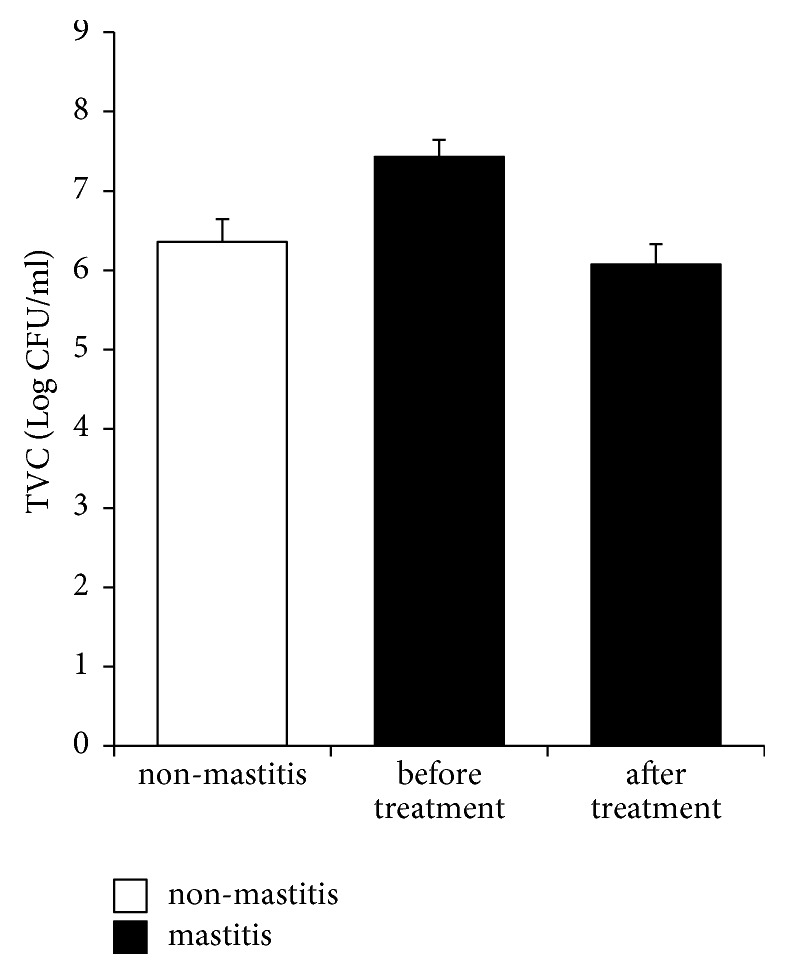
Total viable count (TVC) in milk. Milk samples from non-mastitis and mastitis udder quarters were spread onto TSA plates; bacterial colonies were counted after the plates were incubated at 37°C for 24–48 h. Data are expressed as mean ± SE.

TVC test indicated that our non-mastitis milk (milk from cows with CMT scored N) contained 7.20 × 10^4^ to 2.13 × 10^7^ colony forming units (CFU)/mL (Figure 1). In mastitis milk, the TVC was increased, between 2.21 × 10^6^ and 7.87 × 10^8^ CFU/mL, which was then decreased to 2.10 × 10^4^ – 9.20 × 10^6^ CFU/mL after YXT treatment, close to that in non-mastitis milk (Figure 1).

### 3.4. Clot Was Reduced to Normal Levels after YXT Treatment

**Figure 2:**
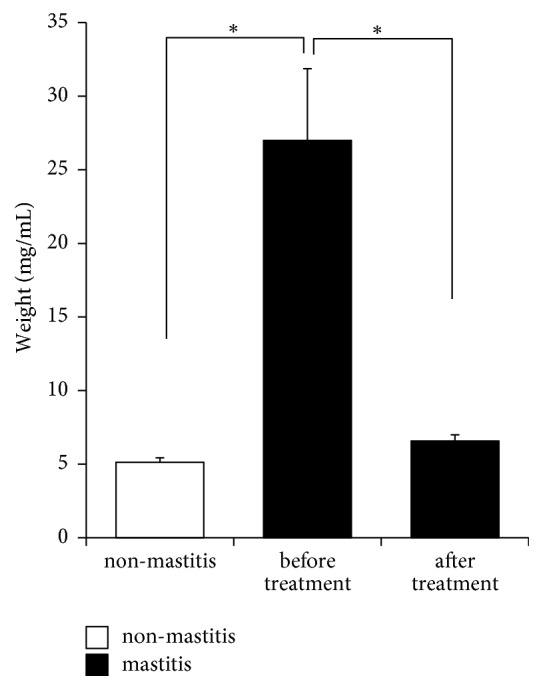
Amounts of clot in milk. Milk samples from non-mastitis and mastitis udder quarters were filtered through filter papers (Advantec Grade No. 1). The clots that remained on filter papers were air-dried and weighed. Data are expressed as mean ± SE, *∗P *< 0.05.

Results showed that non-mastitis milk contained 5.13 ± 0.3 mg/mL of clots (Figure 2). By contrast, milk from udders with distinct or strong mastitis contained as much as 5.3 times more clots (27.0 ± 4.87 mg/mL) than did that from non-mastitis udders; after YXT treatment, the clot content reduced to 6.56 ± 0.43 mg/mL, within the range in non-mastitis milk (Figure 2).

### 3.5. LDH Activity Decreased to Normal Levels after YXT Treatment

**Figure 3:**
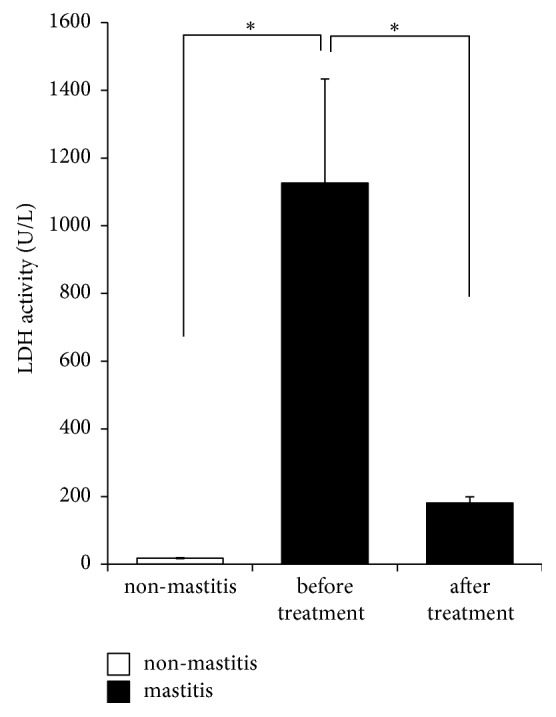
LDH activity in milk. Whey of milk samples from non-mastitis and mastitis udder quarters was obtained by centrifugation and then measured by Roche Cobas Integra 800 biochemistry analyzer. Data are expressed as mean ± SE, *∗P* < 0.05.

In our non-mastitis milk, the LDH activity was 17.88 ± 1.42 U/L (Figure 3). By contrast, the activity increased to as high as 1126.07 ± 307.61 U/L in mastitis milk, 63 times higher than that in non-mastitis milk. After YXT treatment, it decreased to 181.0 ± 18.55 U/L which was about 84% less than that before treatment (Figure 3).

### 3.6. Inflammatory Cytokine Levels Decreased to Normal Levels after YXT Treatment

**Figure 4:**
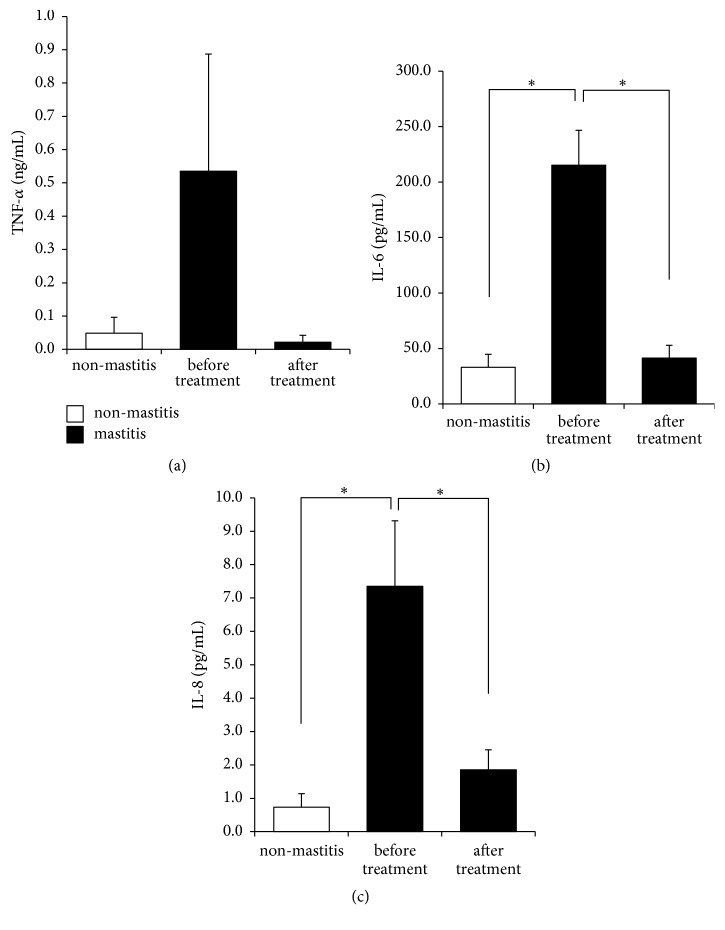
Inflammatory cytokines in milk. Whey was obtained as described in Figure 3. Concentrations of (a) TNF-*α*, (b) IL-6, and (c) IL-8 were measured by the enzyme-linked immunosorbent assay (ELISA). Data are expressed as mean ± SE, *∗P *< 0.05.

TNF-*α*, IL-6, and IL-8 were 0.05 ± 0.05 ng/mL, 33.13 ± 11.68 pg/mL, and 0.74 ± 0.4 pg/mL in our non-mastitis milk, versus 0.54 ± 0.35 ng/mL, 215.3 ± 31.31 pg/mL, and 7.35 ± 1.96 pg/mL in the mastitis milk, respectively, which were decreased to 0.02 ± 0.02 ng/mL, 41.4 ± 11.46 pg/mL, and 1.85 ± 0.6 pg/mL after YXT treatment (Figure 4).

## 4. Discussion

Bovine mastitis is a serious disease causing considerable economic loss worldwide [6, 7], which has a prevalence of 48.6%–86.2% at cow level and 19.6%–55.4% at quarter level on the basis of CMT scores 1, 2, and 3 [1–3]. Our CMT results on 179 lactating cows in a dairy ranch in eastern Taiwan revealed that the prevalence of mastitis, including score 1, 2, and 3, was 54.19% (97/179) at the cow level and 24.53% (170/693) at the udder quarter level. A survey of 17 dairy farms in central Taiwan during August 1992–June 1993 revealed that 691 udder quarters of 992 dairy cows had mastitis on the basis of CMT score 2 and 3, indicating a prevalence of 17.4% (udder quarter level) [29]. Compared with the case in this study, the prevalence of mastitis udder quarters that had a CMT score of 2 or 3 (9.67%, 67/693) is lower than that reported in central Taiwan. Thus, the prevalence of cow mastitis found in Taiwan is within the range, but at the lower end, known worldwide.

Mastitis of lactating cows is commonly treated through intramammary infusion of antibiotic ointments; however, these treatments are costly and are anticipated to become problematic because of wide distribution of the multidrug-resistant causative agents [8, 21]. Therefore, in this study, efforts were made to test herbal extracts as an alternative treatment. Many herbs used in TCM have heat-clearing, detoxifying, anti-inflammatory, and antibacterial activities [23, 24]. Our YXT prepared from two of such herbs,* A. dahurica* and* R. officinale*, which has been shown to have antibacterial and anti-inflammatory activities [25], was the therapeutic agent of choice.

Our TVC test detected 7.20 × 10^4^ – 2.13 × 10^7^ CFU/mL and 2.21 × 10^6^ – 7.87 × 10^8^ CFU/mL for the non-mastitis and mastitis milk, respectively (Figure 1), which are close to the values reported previously, 1 × 10^3^ – 1.34 × 10^6^ CFU/mL versus 8.7 × 10^6^ –3.4 × 10^7^ CFU/mL [30–32]. After YXT treatment, the values were found to decrease to 2.10 × 10^4^ – 9.20 × 10^6^ CFU/mL in mastitis milk, suggesting that YHT contains effective antibacterials. These active antibacterial compounds included at least rhein and emodin as detected in our YXT used.

The inflammatory responses of mastitis at least increase SCC and levels of inflammatory cytokines, including TNF-*α*, IL-6, and IL-8, in milk [15–18]. Milk from our test cows with mastitis showed elevated levels of all these mastitis indicators plus clot content and TVC (Figures 1–4). Our results demonstrate that the levels of all these inflammation indicators in milk reverted to normal level after treatment, suggesting that YXT had anti-inflammatory and immunomodulating activities. These results corroborate previous findings that extracts prepared from* A. dahurica* and* R. officinale* possess these activities, which include the following: (1) the ethanolic extract of* A. dahurica* reduces TNF-*α* and IL-6 production in RAW264.7 macrophages by suppressing the NF-*κ*B pathway [33], (2) emodin, the active compound of* R. officinale*, regulates the expression of inflammatory cytokines in RAW264.7 macrophages by suppressing NF-*κ*B activation [34, 35], (3)* A. dahurica* and* R. officinale* extract reduce TNF-*α* level in LPS-stimulated THP-1 cell [26], and (4) in mice with LPS-induced mastitis, 1 mg/kg emodin can reduce the infiltration of neutrophils and the expression of TNF-*α*, IL-1*β*, and IL-6 in the mammary glands by inhibiting the activation of NF-*κ*B and MAPK signaling pathways [36]. Our results show that our YXT contained emodin, thus supporting its anti-inflammatory and immunomodulating activities.

LDH, considered as a sensitive indicator of bovine mastitis [13, 14], is normally detectable in cow milk at 14.0–485.94 U/L. Its levels in mastitis milk are elevated to 227.07–1524.04 U/L [13, 14, 37, 38]. Notably, the LDH activity detected in our mastitis milk was 63 times higher than that in non-mastitis milk, which was found to decrease to the normal levels (181.0 ± 18.55 U/L) after YXT treatment. Since LDH is released mainly by the damaged udder epithelial cells, disintegrated leukocytes, and invading microorganisms [39], our results thus suggest that in addition to reducing the bacterial cell number, YXT treatment has reduced the number of recruited leukocytes and damaged udder epithelial cells. This notion is reasonably supported by the previous reports indicating that (1)* A. dahurica* polysaccharide accelerates rat skin cell proliferation [40], (2) emodin can aid in maintaining normal mammary gland histopathology and attenuating myeloperoxidase activity (the increase in which reflects the mammary gland injury) in LPS-induced mastitis mice [41], and (3) emodin accelerates cutaneous excisional wound healing and improves tissue reorganization in a rat model [42]. The healing effects of mastitis are thus considered to attribute the polysaccharides and emodin present in our YXT.

Studies have reported the treatment of bovine mastitis through oral administration of herbal decoctions [22]. However, because of the heavy body weight of cows, large amounts of herbs are commonly required for each dose. For example, each dose of the commonly used Pu Gong Ying San contains dried form of 100 g of* T. mongolicum*, 50 g of* V. patrinu*, 50 g of* L. japonica *Thunb., 30 g of* Pericarpium Citri Reticulatae Viride*, and 30 g of* Glycyrrhiza uralensis *Fischer, which are administered daily to each cow [22]. The preparation of such a decoction and its oral administration to 48.6%–86.2% of the cows, which is the average rate of mastitis incidence on a ranch [1–3], would be costly and labor-intensive. By contrast, our method of applying the herbal extract through intramammary infusion is similar to the conventional approach of antibiotic ointment application, and only small amount of YXT is required daily for each cow. Additionally, antibiotic ointments were intramammary therapy for 5 to 8 days [43], whereas our YXT treatment course was 3 days. Thus, the advantages of YXT treatment are as follows: (1) YXT preparation and administration are simple and require inexpensive materials, (2) YXT treatment course is shorter than that of conventional antibiotic therapy, (3) because YXT contains multiple active constituents, exerting a cocktail effect, probability for the causative agents to develop drug resistance might be relatively low.

## 5. Conclusions

Our results indicating the effectiveness of YXT treatment are consistent with the previous findings that* A. dahurica* and* R. officinale* have heat-clearing, detoxifying, anti-inflammatory, and antibacterial activities and help maintain the normal histopathology of the mammary gland in animal models. The possible constitutes in YXT are considered to be emodin, rhein, and polysaccharides, which possess antibacterial, anti-inflammatory, and healing effects. Compared with conventional therapy, YXT treatment needs a short shorter treatment course and might have lower probabilities for the causative agents to develop drug resistance.

## Data Availability

The data used to support the findings of this study are included within the article and have been cited.
